# Drug-induced sleep endoscopy while administering CPAP therapy in patients with CPAP failure

**DOI:** 10.1007/s11325-020-02098-x

**Published:** 2020-05-06

**Authors:** E. Dieleman, C. C. A. F. M. Veugen, J. A. Hardeman, M. P. Copper

**Affiliations:** 1 Department of Otorhinolaryngology Head and Neck Surgery, Sint Antonius Hospital, Koekoekslaan 1, 3435 CM Nieuwegein, The Netherlands; 2 Department of Otorhinolaryngology Head and Neck Surgery, Erasmus University Medical Center, Doctor Molewaterplein 40, 3015 GD Rotterdam, The Netherlands; 3 Department of Otorhinolaryngology Head and Neck Surgery, University Medical Center Utrecht, Heidelberglaan 100, 3584 CX Utrecht, The Netherlands; 4 Department of Pulmonology, Sint Antonius Hospital, Koekoekslaan 1, 3435 CM Nieuwegein, The Netherlands

**Keywords:** Obstructive sleep apnea, Drug-induced sleep endoscopy, Continuous positive airway pressure, CPAP failure

## Abstract

**Study objectives:**

To study the pattern of upper airway collapse in patients with CPAP failure by performing DISE while administering CPAP therapy and to determine the reason for CPAP failure accordingly.

**Methods:**

This observational retrospective study comprised 30 patients diagnosed with OSA and CPAP failure, who underwent DISE while administering CPAP therapy. During DISE, the upper airway was assessed with and without CPAP therapy using the VOTE classification. Additionally, a jaw thrust maneuver was performed, in order to mimic the effect of an additional mandibular advancement device (MAD) in combination with CPAP therapy. Consequently, the outcome of DISE was translated into a clinically relevant categorization.

**Results:**

Eleven patients (37%) had a persistent anteroposterior (AP) collapse, including a collapse at velum, tongue base, or epiglottis level and multilevel collapse. Eight patients (27%) had a floppy epiglottis. Five patients (17%) had a persistent complete concentric collapse (CCC) and three patients had a persistent laryngeal collapse (10%). In three patients (10%), no airway collapse was found after CPAP administration.

**Conclusions:**

Based on the results of the reported study, in most cases, the potential cause of CPAP failure can be determined by this new diagnostic method. Consequently, suggestions can be made for additional therapy.

## Introduction

Obstructive sleep apnea (OSA) is a sleep-related breathing disorder characterized by repetitive partial or complete pharyngeal collapse causing reduction (hypopnea) or cessation (apnea) of airflow resulting in hypoxemia associated with sleep fragmentation, daytime sleepiness, and possible cardiovascular and metabolic dysfunction [1, 2]. OSA is a common condition globally. Population-based studies show that approximately 4% of men and 2% of women are affected by OSA [3]. In a more recent study, prevalence showed to be even higher with approximately 23% of women and 50% of men being affected [4]. Continuous positive airway pressure (CPAP) is unequivocally regarded as the gold standard treatment and often the treatment of first choice in patients with severe OSA. CPAP works as pneumatic splint preventing nocturnal collapse of the upper airway, reducing the apnea-hypopnea index (AHI), and improving the quality of sleep [5, 6]. However, its effectiveness can be limited by adherence and tolerance, insufficient decrease in AHI, and limited improvement of symptoms [7]. In the present literature, the term CPAP failure is being used and interpreted in various ways including both poor tolerance and insufficient decrease in AHI. Recently, in order to create an unambiguous definition, a new nomenclature was proposed: (1) CPAP failure upon efficacy in case of an insufficient decrease in residual AHI above 5 apneas per hour, (2) CPAP failure to diminish symptoms when symptoms remain in spite of an adequate decrease in AHI, (3) CPAP intolerance in case of side effects and/or psychological reluctance, and (4) CPAP non-adherence in case of incorrect or insufficient use of CPAP [2]. Multiple studies have been performed to identify factors that influence or predict CPAP intolerance or non-adherence [7–10]. However, not much is known about predictors for CPAP failure upon efficacy (hereinafter referred to as “CPAP failure”).

Drug-induced sleep endoscopy (DISE), first described in 1991 by Croft and Pringle, is a diagnostic evaluation tool for the degree, level(s), and pattern of upper airway obstruction in patients with OSA [11, 12]. DISE is often performed in order to consider other treatment options like surgical procedures, oral appliance treatment (including mandibular advancement devices), and upper airway stimulation. Recently, various studies have shown that DISE can be successful for CPAP titration; characteristics of airway collapse were evaluated as possible predictors for CPAP titration level [13–15]. However, to date, there are no studies that focus on evaluating the pattern of upper airway collapse while administering CPAP therapy to determine the cause of CPAP failure. In this paper, we give a detailed overview of our findings during DISE and identify the cause of CPAP failure individually. Consequently, suggestions will be made for additional therapy.

## Material and methods

### Study design and population

This study was designed as a retrospective, single-center descriptive cohort study including 30 consecutive patients diagnosed with CPAP failure due to an insufficient decrease in AHI above 5 apneas per hour. All patients were previously diagnosed with OSA which was either confirmed by polysomnography (PSG) or respiratory polygraphy (home sleep apnea test or PG) and were initially treated with CPAP. During follow-up, all patients experienced persistent OSA-related complaints and repeatedly measured an AHI above 5 apneas per hour despite intensive support and additional CPAP titration. Patients were extensively discussed in the multidisciplinary sleep team, composed of ENT-surgeons, neurologists, oral and maxillofacial surgeons, and pulmonologists and were diagnosed with CPAP failure. Drug-induced sleep endoscopy with and without administering CPAP therapy was carried out in order to identify the cause of CPAP failure. Subjects with and without previous nasal and/or pharyngeal surgery were included.

### Drug-induced sleep endoscopy

**Fig. 1:**
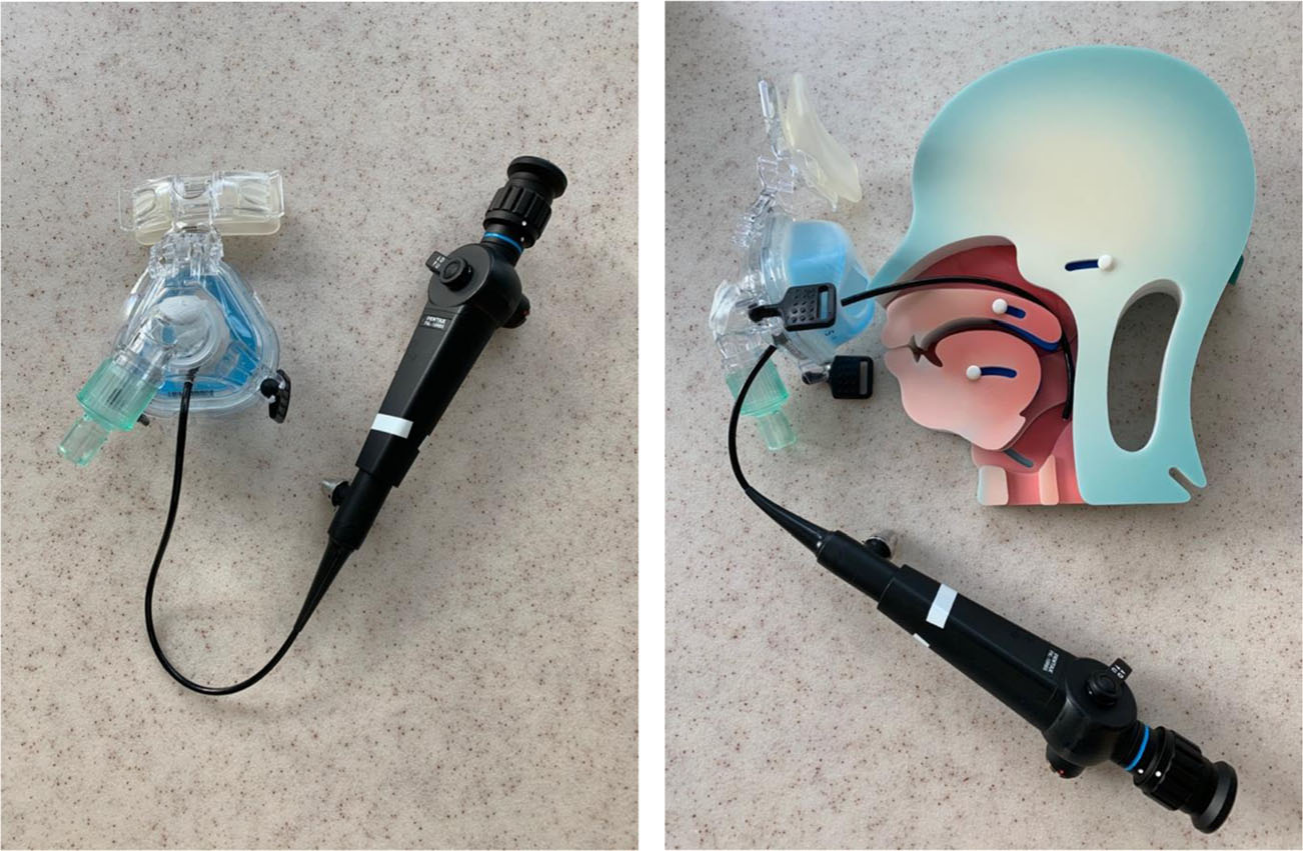
Front and side view of the adapted CPAP mask. The adapted CPAP mask has a small opening in front allowing an endoscope to enter the nose, while permitting to increase CPAP pressures without air leakage

Drug-induced sleep endoscopy was carried out in a quiet operating room with dimmed lights. All procedures were carried out by the same experienced ENT-surgeon (MC) with an anesthesiologist to manage sedation. Sleep was induced by an initial bolus of 1 mg/kg propofol followed by manual titration of propofol. The optimal depth of sedation was reached when the patient began to snore and/or no awakening from vocal or tactile stimuli was achieved. Once a proper level of sedation was approached, the upper airway was thoroughly observed by flexible fiberoptic laryngoscopy. The upper airway was assessed in supine position using the VOTE classification earlier described by Kezirian et al. (Table 1) [16, 17]. This classification system is commonly used to assess levels and structures that may contribute to upper airway obstruction, namely velum (V), oropharynx (O), tongue base (T), and epiglottis (E). For each anatomical level, the configuration (anteroposterior, lateral, or concentric) and severity (no obstruction, partial obstruction, or complete obstruction) of the upper airway collapse were described. Subsequently, an adapted CPAP mask—allowing an endoscope to enter the nose, while permitting to increase CPAP pressures without air leakage—was adjusted (Fig. 1). After the flexible laryngoscope was introduced into the nasal cavity via the adapted CPAP mask, CPAP therapy was started at a pressure of 6 cm H_2_O and gradually enhanced until the potential obstruction was discontinued or a pressure of 16 cm H_2_O was reached. Again, the upper airway was assessed by using the VOTE classification while administering CPAP therapy. Additionally, a jaw thrust maneuver was performed, in order to mimic the effect of mandibular advancement device (MAD) in combination with CPAP therapy. The jaw thrust maneuver was called positive if the obstruction was discontinued on all levels. The jaw thrust maneuver was called negative if the obstruction was still present on one (or more) levels.

**Table 1:**
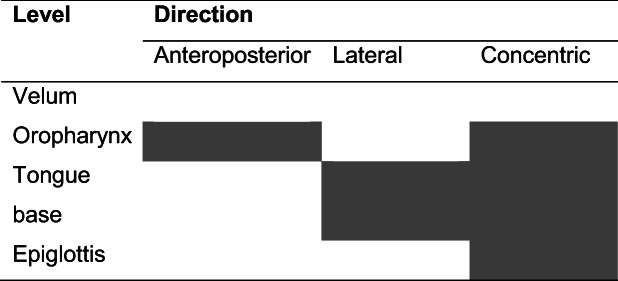
VOTE classification. At each level, the degree and configuration of obstruction should be classified. In each individual, only one degree and configuration of obstruction can be scored on each level. Open boxes reflect the potential configuration that can be visualized related to a specific structure. Shaded boxes reflect that a specific structure-configuration cannot be seen (for example, oropharynx lateral walls in an anteroposterior direction). The degree of obstruction is classified as: 0, no obstruction (no vibration, collapse < 50%); 1, partial obstruction (vibration, collapse 50–75%); 2, complete obstruction (collapse > 75%); x, not assessable

### Statistical analysis

The statistical analysis was performed by using Statistical Package for Social Studies (IBM SPSS Statistics version 24 for Windows, New York, NY, USA). Descriptive statistics were accomplished to create the baseline characteristics. Continuous variables are presented as means with standard deviations. Categorical variables are presented as frequencies with percentages. Comparisons between groups were performed using chi-square test for categorical variables, Student’s *t* test, and univariate analysis of variance (ANOVA) for continuous variables. For all analyses, a two-tailed *p* value of < 0.05 was considered statistically significant.

## Results

### Baseline characteristics

**Fig. 2:**
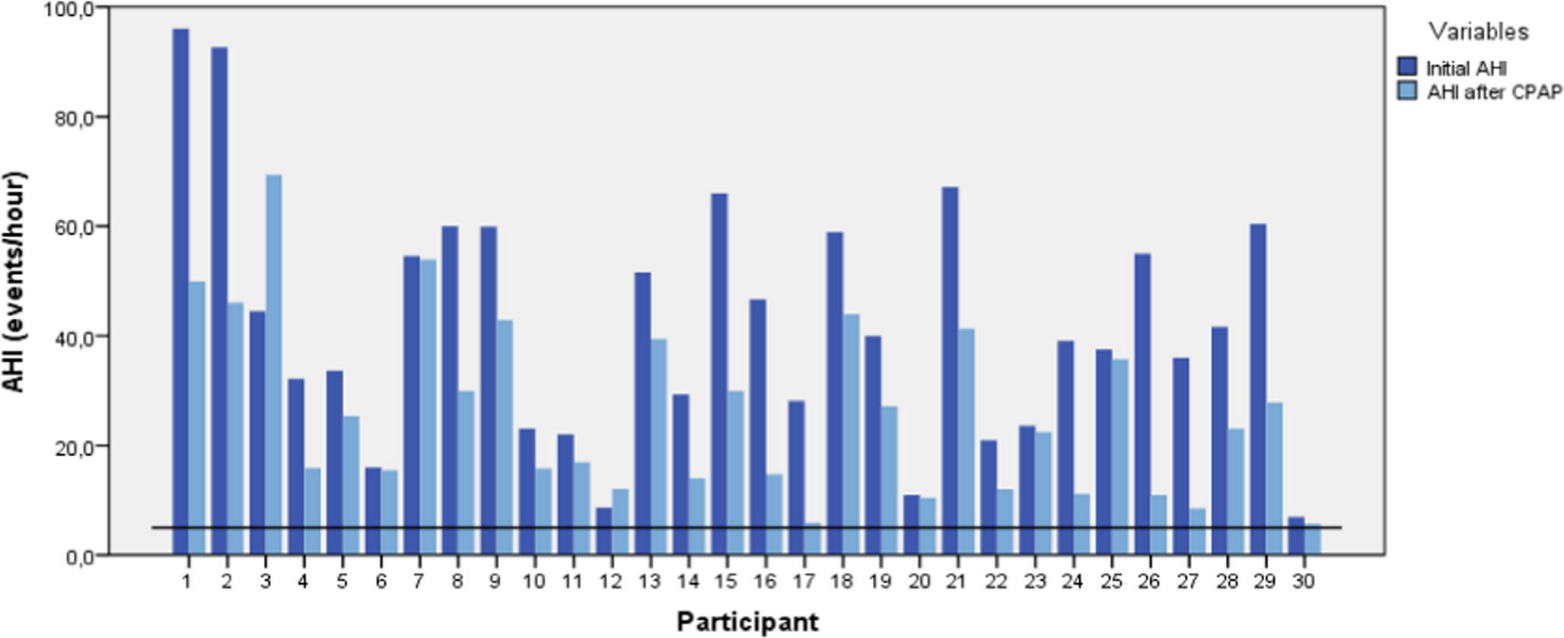
Bar graph showing the pretreatment AHI and AHI with CPAP therapy. Pretreatment AHI is presented in darker blue, AHI with CPAP therapy is presented in light blue. The horizontal black line indicates an AHI of 5 events/h

The study population consisted of 30 patients. Patients were predominantly men (80%), with a mean age of 56.8 ± 13.0 years and a mean BMI of 28.6 ± 4.5 kg/m^2^. Previous nasal/or pharyngeal surgery was performed in 13 patients (43%). In two patients (7%), uvulopalatopharyngoplasty was performed as previous OSA treatment. Nine patients (30%) underwent previous tonsillectomy. However, in all patients, the tonsillectomy was not OSA related. Two patients (7%) underwent previous closure of a cleft palate. The mean pretreatment AHI was 42.1 ± 22.4 (7.0–96.0); the mean central breathing events per hour were 4.4 ± 6.9. The mean AHI with CPAP therapy was 26.0 ± 16.3 (5.7–69.4) (Table 2). The initial AHI and AHI with CPAP therapy are presented for each subject individually in Fig. 2. A significant decrease in AHI after CPAP therapy with an average of 16.1 events per hour was found (95% CI, 10.0–22.3; *p* < 0.0005). However, all patients experienced persistent OSA-related complaints and measured a residual AHI above 5 apneas per hour.

**Table 2:** Baseline characteristics of included patients with CPAP failure

Patient characteristics	Mean ± SD	Range
Men^a^	24 (80%)	–
Age^b^	56.8 ± 13.0	30–78
BMI^c^	28.6 ± 4.5	17.9–38.6
Pretreatment AHI^d^	42.1 ± 22.4	7.0–96.0
AHI with CPAP therapy	26.0 ± 16.3	5.7–69.4

SD, standard deviation; *BMI*, body mass index; *AHI*, apnea-hypopnea index; *CPAP,* continuous positive airway pressure

^a^Gender is expressed as number and percentage (%) instead of mean ± SD

^b^Age in years

^c^BMI in kg/m^2^

^d^AHI in apneas and hypopneas per hour

### DISE analysis

**Fig. 3:**
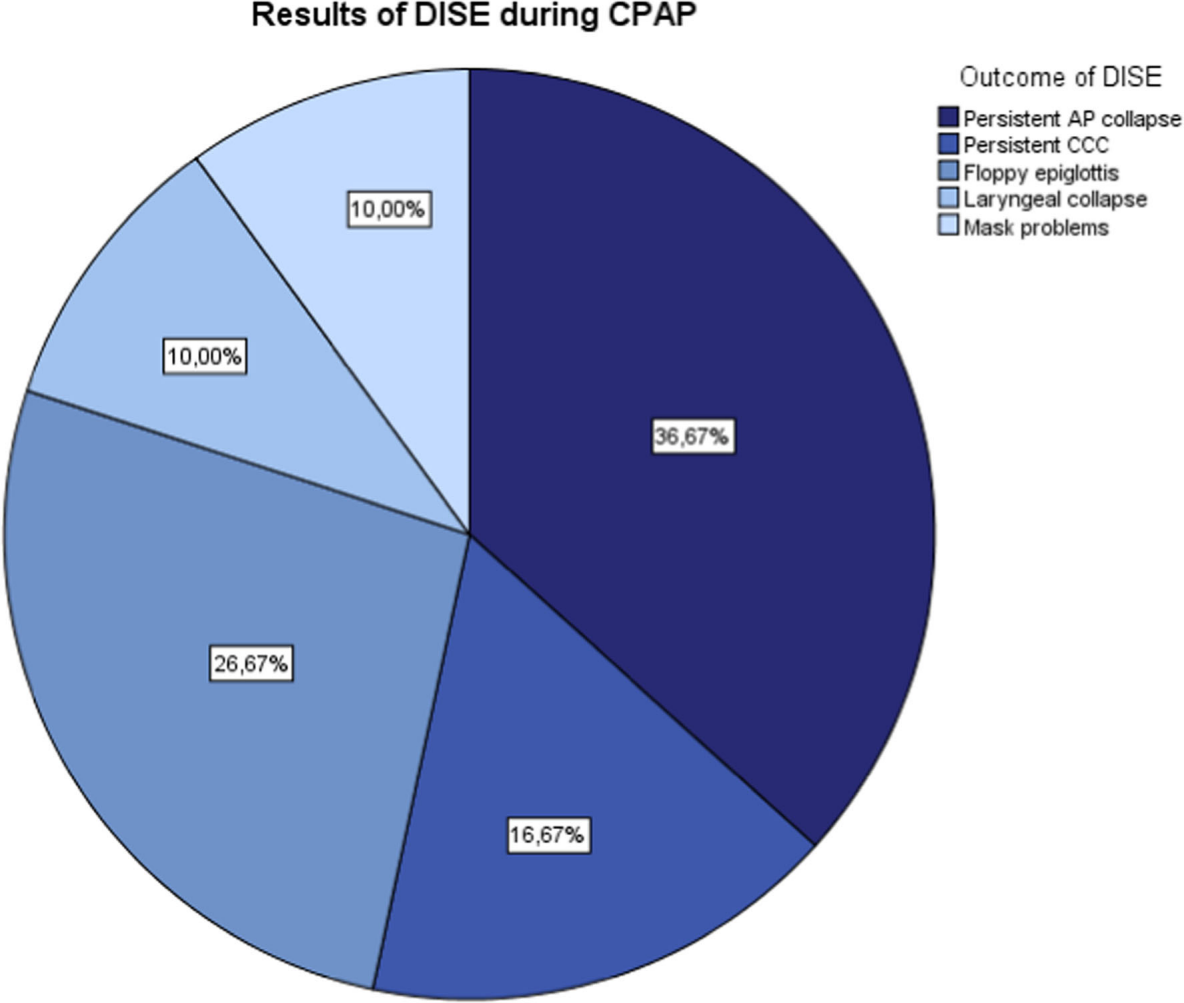
Distribution of outcome of the examination during DISE when CPAP therapy is administered

All patients underwent DISE, while CPAP was administered, during which no complications occurred. Table 3 shows DISE findings while administering CPAP therapy utilizing the VOTE classification. Figure 3 shows the distribution of the main outcomes. Eleven patients (37%) had a persistent anteroposterior (AP) collapse, including a collapse at velum, tongue base, or epiglottis level and a multilevel collapse. Eight patients (27%) had a floppy epiglottis. Five patients (17%) had a persistent complete concentric collapse (CCC) and three patients showed a laryngeal collapse (10%). In three patients (10%), no airway collapse was found after CPAP administration; in two patients, air leakage via the mouth was objectified, when closing the mouth CPAP therapy was effective. While administering CPAP therapy, a jaw thrust maneuver was performed. The obstruction was discontinued on all levels in 22 cases, two cases showed a persistent obstruction on one (or more) levels. In the three patients who did not have any upper airway collapse while administering CPAP, the jaw thrust maneuver was not performed. In the remaining three patients a jaw thrust maneuver was not reported.

**Table 3:**
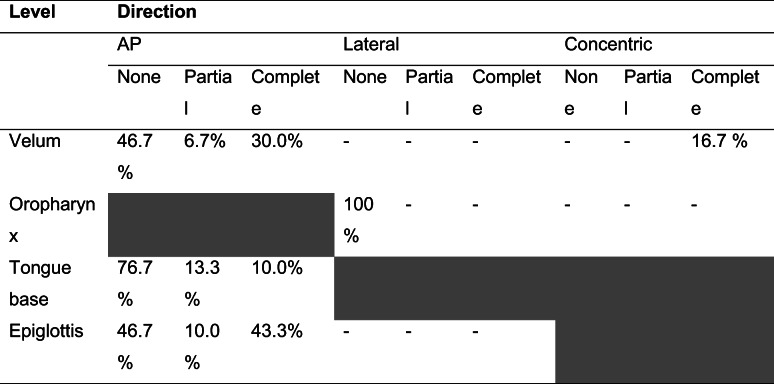
Overview of the distribution of the levels and pattern of upper airway collapse during DISE with CPAP

The mean age, BMI, and AHI with CPAP therapy were compared between the different DISE outcomes. All variables were statistically significantly different for the different outcomes of DISE (*p* = 0.060, *p* = 0.019, and *p* = 0.017 respectively). An LSD post hoc test was done to specify between which groups the underlying difference was statistically significant. Firstly, patients with a persistent CCC were significantly younger than patients with a floppy epiglottis (*p* = 0.043) or a laryngeal collapse (*p* = 0.027). Patients with a laryngeal collapse were significantly older than patients encountering mask problems (*p* = 0.031). Secondly, patients with a persistent CCC had a significantly higher BMI than patients with a persistent AP collapse (*p* = 0.011) or floppy epiglottis (*p* = 0.002). Lastly, comparing the groups based on their mean AHI with CPAP therapy, we found that patients with a persistent AP collapse had a significantly lower AHI than patients with a persistent CCC (*p* = 0.015) or laryngeal collapse (*p* = 0.026). Patients with mask problems had a significantly lower AHI compared to patients with persistent CCC (*p* = 0.007) or laryngeal collapse (*p* = 0.010).

### Treatment

**Fig. 4:**
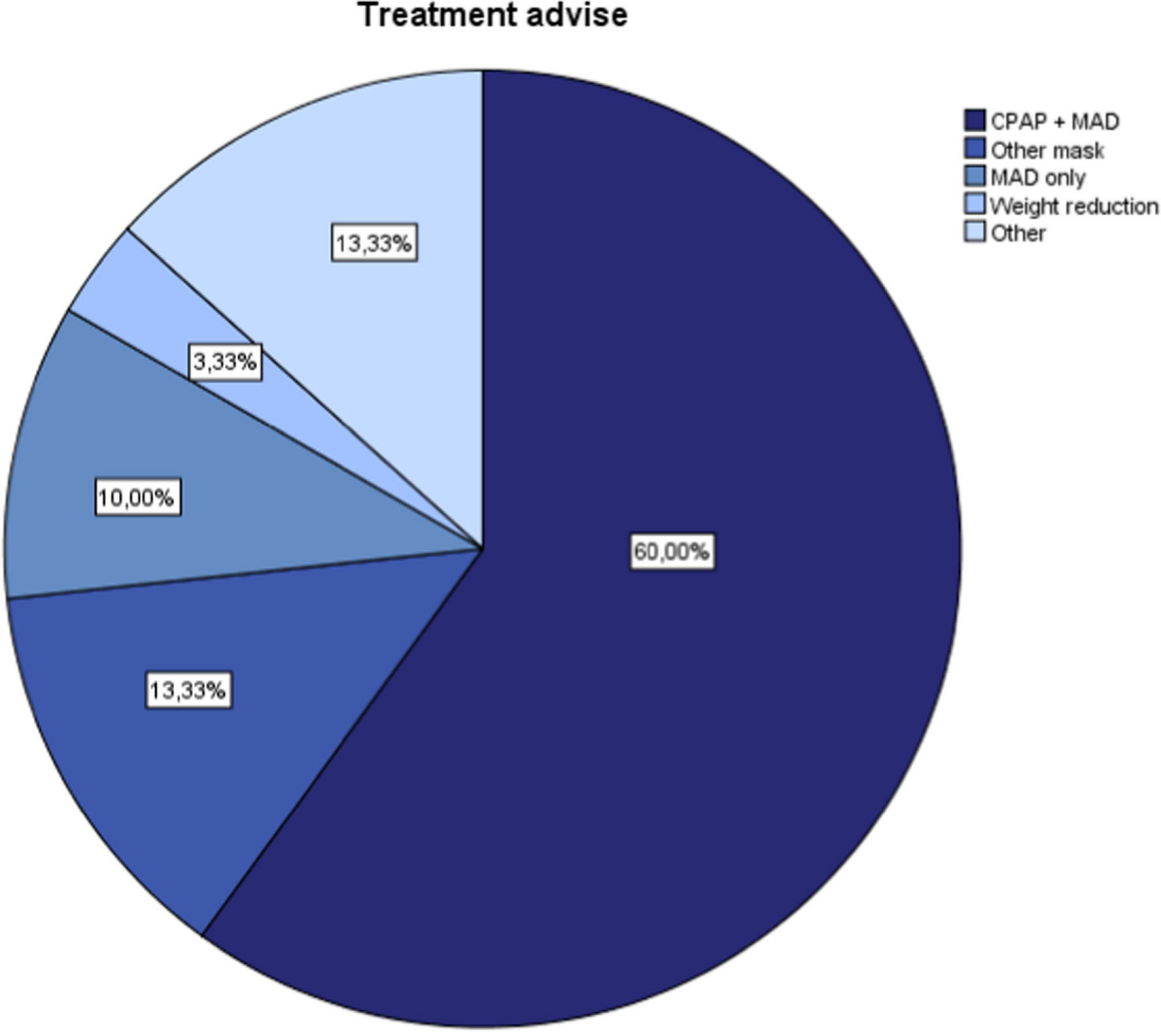
Distribution of the different treatment options advised on account of the DISE outcome

The distribution of different treatment options advised on account of the DISE outcome is shown in Fig. 4. In 21 of the 22 patients with a positive effect of the jaw thrust maneuver, either a MAD or a MAD combined with CPAP therapy was advised. In the other patient, a therapy including a MAD was impossible due to an edentate mandible. In four patients, a different CPAP mask was advised. One patient was advised to lose weight. In four patients, other treatment options were advised, including soft palate surgery, referral to a plastic surgeon because of CPAP failure due to an earlier unsuccessful operation of a cleft palate, and tracheotomy because of CPAP failure due to a laryngeal collapse at the level of the arytenoid cartilages.

## Discussion

In this study, we were able to observe the effect of CPAP therapy on the pattern of upper airway collapse. Our findings show that a possible reason for CPAP failure can predominantly be identified. Previously, studies have been conducted to expand the utilization of DISE for CPAP titration and to evaluate possible predictors for CPAP titration level [14, 15]. Additionally, studies have been conducted to evaluate the upper airway collapse in patients with CPAP failure [18, 19]. However, to date there are no studies that focus on evaluating the pattern of upper airway obstruction while administering CPAP therapy. Acknowledging this problem, we believe that our results are highly relevant to the field. To the best of our knowledge, this is the first study to investigate the role of DISE while administering CPAP therapy as a method to clarify possible mechanisms leading to CPAP failure and to address further treatment options. Our results may extent the utilization of DISE as a new diagnostic method in patients with OSA and CPAP failure.

In 2017, Torre et al. have shown that CPAP has a greater impact on the lateral walls of the oropharynx than it has on the velum, tongue base, or epiglottis [15]. A result that is endorsed by a study of Schwab et al. [20]. Our findings underline these observations since persistent lateral collapse of the oropharynx was not observed in our study. Another interesting finding is that some patients who were shown to have a floppy epiglottis during CPAP treatment were satisfied with this treatment for many years. This raises the question if this phenomenon might be instigated by long-term CPAP usage. Furthermore, in the present study, persistent CCC was associated with a higher BMI. The same observations were previously made by Hasselbacher et al. and Steffen et al. [19, 21]. Additionally, patients with persistent CCC or laryngeal collapse had a higher AHI with CPAP therapy than patients with AP collapse. Previous authors have shown similar results; patients suffering from CCC have a significantly higher initial AHI [18, 19, 21]. In a recent study that focuses on DISE as a selection tool for upper airway stimulation by Vanderveken et al., it is stated that the absence of CCC can predict success of upper airway stimulation [22]. Our results indicate that, possibly, CCC is also a negative predictor for CPAP therapy. However, the sample size in our study was limited, so further investigations with larger sample sizes need to be conducted in the future to validate these results.

### Limitations

This study has several limitations. It is obvious that assessment of the upper airway during DISE is based on subjective findings and therefore, prone to experience bias. However, previous studies have shown a moderate to substantial interrater reliability depending on the experience of the surgeon [23, 24]. Furthermore, the degree of anesthetic depth and body position can alter the upper airway collapse [25, 26]. Opponents of DISE argue that pharmacologically induced sleep, e.g., by propofol as in this study, is characterized by changing sleep patterns. Conversely, Rabelo et al. have shown that the AHI and other respiratory parameters remain unaffected [27]. It is nevertheless important not to oversedate [25]. Patients respond differently to propofol; therefore, it is stressed that the technique to elicit sleep must be standardized rather than to establish a universal concentration for all patients [27]. In this context, we used a consistent method of sedation in all patients by administering an initial bolus of 1 mg/kg. However, after the initial bolus, titration of propofol was administered manually until the patient began to snore and/or no awakening from vocal or tactile stimuli was achieved. In order to aim for a standardized technique, in future measuring sedation depth and the use of target-controlled infusion pumps should be considered. It may also be discussed that the jaw thrust maneuver to mimic the effect of a MAD is a very imprecise maneuver as it lacks reproducibility and standardization. However, despite its limitations, performing a jaw thrust maneuver can easily and routinely be implemented during DISE and might improve patient selection for (additional) MAD treatment [28]. Undoubtedly, the retrospective nature and the small sample size of this study are the limitating factors. Testing for possible associations between the outcome of DISE and the befitting treatment was not applicable due to this small sample size. Additionally, the correlations found between DISE results and age, BMI, and AHI with CPAP therapy are based on a small sample size and therefore only tentative conclusions can be drawn. Studies with a larger sample size need to be conducted in the future in order to validate these results.

## Conclusion

The results of this study provide important insight into the possible patterns of upper airway obstruction while administering CPAP therapy. In most cases, a possible cause of CPAP failure can be identified individually. Furthermore, we demonstrate that determining the reason of CPAP failure can lead towards new suggestions for treatment options in addition to/instead of CPAP therapy, including surgery or an MAD. However, further analysis of the association between outcome of DISE and treatment options and analysis of the treatment outcome needs to be conducted in future studies.

## Abbreviations

AHI: apnea-hypopnea index
ANOVA: univariate analysis of variance
AP: anteroposterior
CCC: complete concentric collapse
CPAP: continuous positive airway pressure
DISE: drug-induced sleep endoscopy
ENT: ear, nose, and throat
MAD: mandibular advancement device
OSA: obstructive sleep apnea
PG: respiratory polygraphy
PSG: polysomnography
